# Bacteriophages for the Targeted Control of Foodborne Pathogens

**DOI:** 10.3390/foods12142734

**Published:** 2023-07-18

**Authors:** Emmanuel W. Bumunang, Rahat Zaheer, Dongyan Niu, Claudia Narvaez-Bravo, Trevor Alexander, Tim A. McAllister, Kim Stanford

**Affiliations:** 1 Department of Biological Sciences, University of Lethbridge, Lethbridge, AB T1K 1M4, Canada; emmanu-el.bumunang@uleth.ca; 2 Agriculture and Agri-Food Canada, Lethbridge Research and Development Centre, Lethbridge, AB T1J 4B1, Canada; rahat.zaheer@agr.gc.ca (R.Z.); trevor.alexander@agr.gc.ca (T.A.); tim.mcallister@agr.gc.ca (T.A.M.); 3 Faculty of Veterinary Medicine, University of Calgary, Calgary, AB T2N 1N4, Canada; dongyan.niu@ucalgary.ca; 4 Food and Human Nutritional Sciences, Faculty of Agricultural & Food Sciences, University of Manitoba, Winnipeg, MB R3T 2N2, Canada; claudia.narvaezbravo@umanitoba.ca

**Keywords:** food production system, food safety, foodborne disease, bacteriophage, single-cell variants

## Abstract

Foodborne illness is exacerbated by novel and emerging pathotypes, persistent contamination, antimicrobial resistance, an ever-changing environment, and the complexity of food production systems. Sporadic and outbreak events of common foodborne pathogens like Shiga toxigenic *E*. *coli* (STEC), *Salmonella*, *Campylobacter*, and *Listeria monocytogenes* are increasingly identified. Methods of controlling human infections linked with food products are essential to improve food safety and public health and to avoid economic losses associated with contaminated food product recalls and litigations. Bacteriophages (phages) are an attractive additional weapon in the ongoing search for preventative measures to improve food safety and public health. However, like all other antimicrobial interventions that are being employed in food production systems, phages are not a panacea to all food safety challenges. Therefore, while phage-based biocontrol can be promising in combating foodborne pathogens, their antibacterial spectrum is generally narrower than most antibiotics. The emergence of phage-insensitive single-cell variants and the formulation of effective cocktails are some of the challenges faced by phage-based biocontrol methods. This review examines phage-based applications at critical control points in food production systems with an emphasis on when and where they can be successfully applied at production and processing levels. Shortcomings associated with phage-based control measures are outlined together with strategies that can be applied to improve phage utility for current and future applications in food safety.

## 1. Introduction

**Figure 1:**
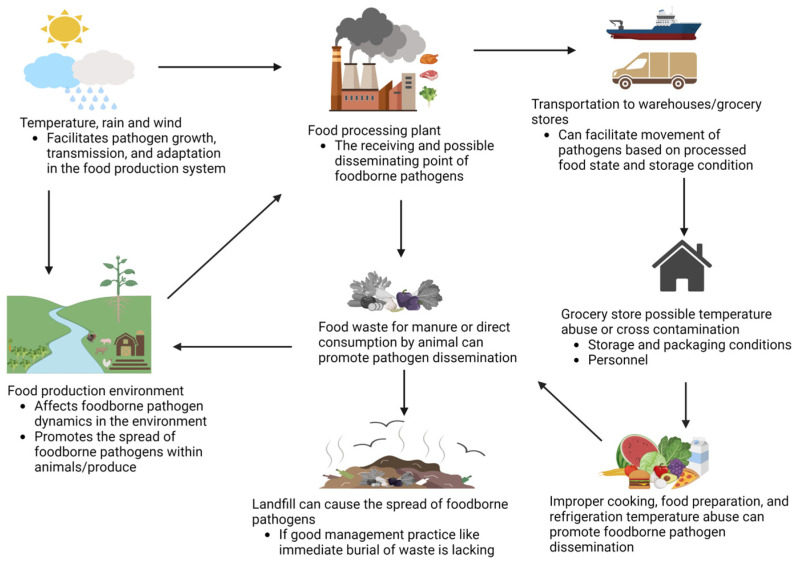
Factors that influence the risk of transmission of foodborne pathogens into the food chain.

The number of foodborne infections is ever-increasing despite legislative and microbiological prevention strategies to ensure food safety and public health. Emerging pathotypes, sanitizer-resistant microbes, and interconnections along the supply chain, such as production environment, contaminated processing equipment/surfaces, and asymptomatic food handlers, are considered major challenges contributing to an increase in foodborne infections [1]. Food-producing animals (cattle, swine, and chickens), leafy greens, soil, and water are the main bacterial sources of foodborne infections. The risks of transmission of foodborne diseases are influenced by environmental conditions such as temperature, moisture, and various agricultural and industrial practices (Figure 1).

Shiga toxigenic *E*. *coli* (STEC), *Salmonella*, *Campylobacter*, and *Listeria monocytogenes* are recognized as the most urgent threats in food-producing systems and are frequently associated with foodborne disease outbreaks. Recent examples include a *Listeria* outbreak in South Africa which recorded 728 patients and 193 mortalities [2]. The case–control and trace-back analysis suggested that polony, a ready-to-eat processed meat, was the source of infection [2]. In Canada, an *E*. *coli* O157 outbreak linked to romaine lettuce was reported and traced to the Yuma growing region in the United States [3]. This outbreak recorded eight cases of illnesses with no deaths. In China, a *Salmonella* outbreak was reported in Changzhi County [4]. *Salmonella* recovered from this outbreak was associated with a multidrug-resistant strain, resulting in 11 cases of illness but no deaths. In England, a *Campylobacter* outbreak was linked to the consumption of raw milk in 2016 [5], affecting sixty-nine individuals with no mortalities.

Various decontamination strategies such as the use of chemical sanitizers (sodium dichloroisocyanurate, quaternary ammonium compounds, chlorine, peracetic acid, and lactic acid), heat treatment (pasteurization), washing (water), and chilling are commonly used during food processing to reduce the risk of pathogens entering the food chain [6,7]. Bacteriophage-based interventions are being implemented as an additional hurdle or in synergy with these current interventions. For example, ListShield™ is a cocktail of bacteriophages that targets *L*. *monocytogenes* and is the first purified phage food additive used commercially to control this pathogen in ready-to-eat meat and poultry products and in food processing environments [8]. SalmoFresh^TM^, specific for *Salmonella,* can be used directly on poultry, fish and shellfish, and fresh and processed fruits and vegetables [9]. A comprehensive list of commercial bacteriophage (phage) products used for the biocontrol of foodborne pathogens in various foods has been reviewed [10]. These commercial phages are “customized combinations” that are of a narrow spectrum, given the vast genetic diversity of foodborne pathogens in different environments and food types. Regardless, the application of phages as biocontrol agents is promising in the food production system.

In this article, we review factors impacting cross-contamination, such as occurrence, prevalence levels, infective dose and persistence associated with foodborne pathogens, and the critical control points within the food production system where the risk of contamination is likely highest, with a focus on where and how bacteriophages can be successfully applied at the food production and processing levels.

## 2. Factors Impacting Cross-Contamination of Pathogens Occurrence, Virulence, and Pathogenicity

Shiga-toxigenic *E. coli* O157 (STEC) super-shedding incidents, where some cattle within a herd can shed >10^4^ CFU/g in feces [11], can contribute to a high prevalence of the pathogen in production farms and subsequently in processing facilities. Compounded by a low infective dose (10–100 cells), STEC infections can cause life-threatening diseases such as hemolytic uremic syndrome (HUS), which can lead to kidney failure, especially in pregnant women, children, the elderly, and the immunocompromised [12]. STEC strains can survive harsh food processing conditions and serve as hot spots for persistent cross-contamination events in food processing environments. Although STEC O157:H7 has been the predominant serogroup associated with HUS worldwide, cases with non-O157 serogroups such as O26, O111, O103, O121, O45, and O145 have also been increasingly associated with foodborne illness, accounting for about 83% of all STEC infections in humans from 2000 to 2010 in the United States [13]. STEC strains can grow over a pH range of 4.4–10.0 [14] and temperatures ranging from 6 to 40 °C [15]. Some strains are heat-tolerant [16], whereas others have demonstrated strong biofilm-forming abilities on food processing surfaces like stainless steel [17,18] and polystyrene [19]. Thus, STEC O157 and a variety of non-O157 STEC strains are pathogens of priority, for which there is an urgent need to develop control strategies to reduce or completely prevent their adulteration of the food chain.

*Listeria monocytogenes*, the causative agent of human listeriosis, is associated with bacteremia, endocarditis, and infection of the central nervous system [20]. *L*. *monocytogenes* is a ubiquitous pathogen that thrives in diverse environments such as soil, water, various food products, and in the digestive tract of humans and animals [21,22]. It can survive and grow over a wide range of temperatures (2–45 °C) and pH (4.6–9.5) and in the presence of high salt concentrations [23,24]. Because of its ubiquitous distribution and robustness, there is a high chance that naturally persistent strains can be introduced into the food processing system through cross-contamination. Additionally, the ability of *L*. *monocytogenes* to form biofilms enables them to survive and persist on food processing surfaces [25]. The infectious dose of *L*. *monocytogenes* is not known but is likely lower in susceptible individuals like immunocompromised patients, the elderly, infants, and pregnant women than in healthy individuals [26]. In addition, *L*. *monocytogenes* may be a transient resident in the gastrointestinal tract of humans, with about 2–10% of human carriers lacking clinical symptoms [27]. These assertions are in line with *L*. *monocytogenes* outbreaks, during which the immunocompromised, neonates, and pregnant women are most likely to be infected [2,23,28].

*Campylobacter jejuni* is one of the causative agents of human campylobacteriosis characterized by mild diarrhea to serious conditions like bloody diarrhea, myocarditis, celiac disease, acute cholecystitis, and colorectal cancer [29]. Poultry meat contaminated with *C*. *jejuni* is the main route of infection in humans [30,31]. Unlike *L*. *monocytogenes*, which is stress resistant, *C*. *jejuni* is very susceptible to environmental stressors such as cold temperatures and acidic (pH of <4.9) conditions [31]. While these susceptibilities could be helpful in limiting the survival of *C*. *jejuni* and thus reducing the chances of cross-contamination, it has a high prevalence in poultry. *C*. *jejuni* is part of the normal microflora in the gastrointestinal tract of poultry, where prevalence can be 100% in broiler chickens at slaughter [32,33]. Furthermore, *C*. *jejuni* numbers are high (10^5^–10^8^ CFU/mL) in slaughterhouse carcass water [31], suggesting that cross-contamination of carcasses is inevitable. Additionally, *C*. *jejuni* may be well protected from harsh environmental conditions in biofilms [34]. The infectious dose of *C*. *jejuni* ranges from 400 to 10^6^ cells in healthy young individuals and is strain-specific [35]. However, the infectious dose may be lower in susceptible/immunocompromised individuals with underlying conditions. Therefore, measures to reduce *C. jejuni* in processing plants are imperative to prevent human disease.

*Salmonella* Typhimurium, the causative agent of salmonellosis, is characterized by various clinical diseases such as gastroenteritis, enteric fever (typhoid fever), and bacteremia [36]. *S.* Typhimurium is a microorganism that thrives at a neutral pH. However, *S.* Typhimurium is capable of tolerating acidic (pH 3.3) conditions [37]. *S.* Typhimurium is widespread in nature and associated with cross-contamination in diverse food sources such as poultry [38], pork [39], beef [40], and fruits and vegetables [41]. The association with multiple food sources may enable it to circulate between different hosts, the environment, and humans through close contact or poor hygienic conditions during food handling. *Salmonella* can survive in low-moisture foods such as powdered milk, chocolate, peanut butter, infant foods, cereal, and bakery products over extended periods of time [42]. *Salmonella* is known to form biofilms on glass slides [43] and stainless steel [44,45]. The ability to form biofilms enhances the ability of *Salmonella* to survive in food-processing plants. The infectious dose of *Salmonella* is estimated to be 100 cells or higher [46] and can vary depending on the susceptibility of individuals.

Overall, it appears that high microbial load, persistence, infectious dose, and risk groups (susceptible individuals in the population) associated with the different pathogens directly correspond to cross-contamination events in the food production system. Although phenotypic variations such as the occurrence of persister cells within biofilms can impede control measures at any stage from production through to the processing plant, it is important to examine the random phenotypic diversity within a homogeneous single-cell population [47,48,49]. Phenotypic heterogeneity in a single-cell clonal population is known to confer resistance to antibiotics [50,51] as a result of the occurrence of two distinct subpopulations, a condition referred to as bi-stability [52]. These two subpopulations help ensure the survival of the population during environmental changes, a condition known as bet-hedging [53]. Therefore, to reduce the number of bacterial pathogens in the food production and processing environment, the possibility of single-cell phenotypic variants throughout the processing environment must be considered.

## 3. Bacteriophages

Bacteriophages (phages) are viruses that infect and kill bacteria. Some phages are host-specific, whereas others can infect multiple species or bacterial genera [54]. The host range of a phage determines the phage–host interaction events such as attachment, infection, and lysis, traits which are important in their application against target pathogen(s). For example, a narrow-spectrum phage is preferred for the control of a target pathogen in a live animal to avoid affecting commensal beneficial bacteria. In contrast, in food processing plants, a phage with a broad host range is preferred to efficiently reduce or eliminate the diversity of strains within a pathogenic species. It has been shown that under suitable conditions, a broad-spectrum phage can revert to a narrow state and vice versa [55]. Therefore, a cautious approach is required to delineate the phage host range [56], as well as employing phages with a broad or narrow host range for specific biocontrol applications.

Phages are ubiquitous in nature, have been isolated from food products like yogurt and cheese [57], and are naturally found in the human gut [58]. This suggests that humans encounter phages either directly or indirectly in daily life. Moreover, oral administration of phages is not known to cause disease in animals [59,60]. Also, because phages are ubiquitously distributed, they can provide an important diverse repertoire of control agents which can be exploited for control of pathogens in environments that possess differing selective pressures like temperature, salinity, pH, and UV radiation.

Lysogenic phages infect bacterial hosts, can integrate into the host genome, and alter the genotypic and phenotypic characteristics of the host. For example, the spread of Shiga toxin by *E*. *coli* O157:H7 is associated with a lambdoid bacteriophage [61], in which the toxin is only expressed when the lysogen becomes lytic during a stress response [49,62]. Lysogenic phages (lambdoid phage) are also being explored for phage-based DNA vaccine delivery in humans [63,64].

Lytic phages infect and kill their bacterial host through lysis. There is an ever-growing interest in lytic phages as control agents against targeted foodborne pathogens, including, *Listeria*, *Salmonella*, *Campylobacter*, and STEC [65,66,67,68]. The increasing interest in lytic phages as biocontrol agents follows the growing concerns with antibiotic-resistant bacteria [69,70] and an increased number of studies that are demonstrating the efficacy of phage therapy. Some of these are reflected in the recent increase in the number of phage clinical studies [71,72], along with the increased sequence-based characterization of phages through comparative genomics and proteomics [73,74,75].

Other than the biological traits of the phage, such as lysogenic or lytic capacity, various environmental factors may affect phage efficacy and viability. For example, external environmental conditions such as UV radiation, pH, temperature, and salt concentration can affect phage activity [76,77,78,79,80,81]. The level of inactivation depends on the intensity of these factors and may differ for different phages. For example, Iriarte, Balogh, Momol, Smith, Wilson, and Jones [76] found that phage populations associated with the surface of tomato leaves plummeted during the months when levels of UV radiation were the highest. Also, the activity of a lambdoid phage (λ-gt11) was reduced at extreme pHs of 11.8 or 2.0 but remained active after 24 h of exposure to pHs of 3–11 [82]. In contrast, the activity of a myovirus, JD007, was completely lost at a pH of 4 after 2 h of exposure at room temperature [83]. Some phages can survive temperature shifts; ≥75% of phages isolated from hot springs in California remained viable at 0 °C, whereas 18 to 30% remained intact at 105 °C [84]. Variations in phage viability are likely driven by the high genetic diversity among phages [81], geographic location, and experimental conditions. Therefore, getting a phage with the desired stable characteristic for target interventions requires both in vitro and field testing. Nevertheless, tailed phages such as T4 and T7 may be preferred, as shown by Ackermann et al. [85], as they remain stable over long periods (10–32 years) of refrigerated storage.

Phage–host exposure can result in phage-insensitive isolates [86]. However, it is possible that phage-insensitive strains are endemic within target pathogen populations as a result of previous exposure to environmental selective pressures. Phenotype-associated subpopulations, referred to as the phenotypic switch, have been reported in *L*. *monocytogenes* exposed to hostile environmental conditions [87]. The presence of phage-insensitive strains within a population is an important consideration for the development of targeted interventions in the phage arms race against foodborne pathogens.

## 4. Food-Production Animals, Leafy Green Produce, and Foodborne Pathogens

Food-production animals and plants are adapting to ever-changing environmental conditions like temperature, moisture, and availability of nutrients. The same applies to foodborne pathogens in the broader environment and in the gut of animals. Temperature, moisture, and availability of nutrients can facilitate pathogen growth, adaptation, and transmission to food-production animals through the food they eat or by wind, contaminated water, or when manure is used as a fertilizer to produce leafy greens. Unlike broad-spectrum antibiotics, which can be administered empirically [88,89] to control pathogens, no single phage preparation can control the entire range of pathogens that can potentially adulterate food. The formulation of phage cocktails has been proposed to be one possible solution to overcoming the limitation of a narrow host range [90]. However, developing a phage cocktail with consideration of the different mechanisms that can influence receptor binding to targeted cells requires an in-depth knowledge of phage diversity [91], as antagonistic interference can impact cocktail efficacy. Also, the phage latent period, the time required for a phage to induce host cell lysis [92], is important for cocktail concoction as a differential infection cycle of phages within a cocktail may select for the progeny of phages with the shortest latent period in the presence of a target pathogen defying the goal of the cocktail. As a result, even though phages offer the potential to control foodborne pathogens, optimizing phages for empiric application in livestock and food processing environments can be difficult and may require substantial research and development efforts. Therefore, target control of a pathogen would be highly preferred, with formulated cocktails possibly reducing the emergence of phage-resistant bacterial mutants as a result of their ability to target multiple receptors in pathogens [90].

### 4.1. Application of Phages for Target Control of Foodborne Pathogens in Food-Production Animals

**Figure 2:**
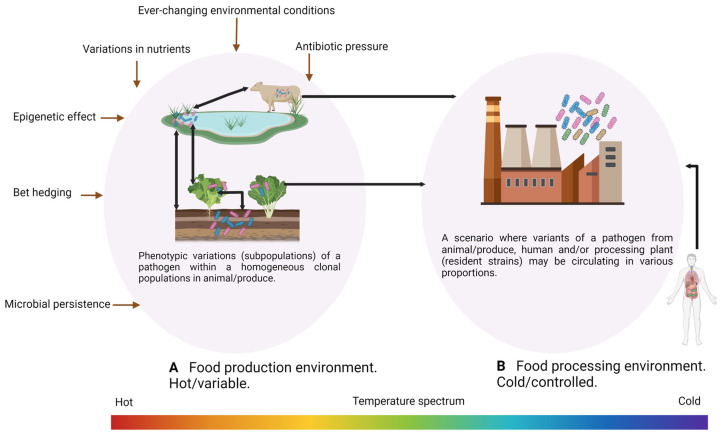
(**A**) Single-cell heterogeneity within food-production animal/produce (**B**) and a diverse pathogen population in processing environments. These diverse populations may arise due to response to environmental selective pressures and from cross-contamination events.

**Figure 3:**
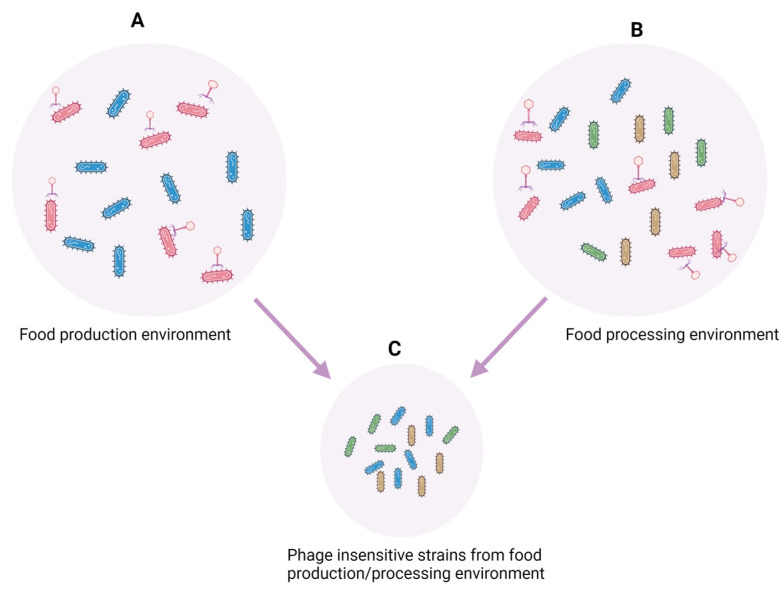
(**A**) Phage interaction with phenotypic variants of a pathogen within food-production animal/produce; (**B**) phage treatment of a pathogen population in a processing environment; and (**C**) combining pathogen populations on animals/crops with that in food processing environments after phage treatment may result in phage-insensitive strains.

The prevalence and persistence of foodborne pathogens on the hide and within the gut of food-producing animals on farms (pre-harvest) is the primary source of cross-contamination in the food chain. On-farm phage-based control strategies can successfully reduce target pathogen populations on and within livestock before slaughter [93,94]. Incidence and persistence of a target pathogen can vary among various animals, animals within a herd, or in different sites in the same animal. For example, *C. jejuni* is known to be prevalent in broiler chickens at slaughter, with all birds being carriers on some farms [32,33]. In cattle herds, up to 20% of the animals have been identified as *E. coli* O157 super-shedders [11,95]. Differentiating super-shedding cattle within a herd from transient shedders can be challenging and may require substantial sampling events over a long period [96] before treatment with phages. Various environments within the cattle gut, such as the rumen, cecum, colon, and rectum, may be colonized by a target pathogen, with the site within the digestive tract influencing the efficacy of phage-based interventions. Rumen physiological conditions such as pH can inactivate phages [97], whereas pathogen colonization and biofilm formation at the rectum may shelter targeted cells from phages [98]. Even though phage encapsulation has been shown to protect phages at a pH of 3.0 for 20 min of exposure [99], an in-depth understanding of the factors that affect phage stability from storage to delivery is required [100]. Individual phages or cocktails need to be optimized to infect targeted pathogens across various environments and in biofilms. Also, phenotypic variants which may arise within the target microbial population due to responses to environmental pressures in food-producing animals (Figure 2) can further compromise phage-based control strategies. Some subpopulations within the target population may survive phage treatment (Figure 3). The use of comparative genomics can differentiate, track, and provide valuable insight into plausible cell variants within a targeted pathogen population before phage application. This approach can also be used to select phages or concoct a phage cocktail with a broad host range to include phenotypic variants as part of the targeted pathogen population.

Several studies (*n* = 15) have explored the potential of phages to control *E. coli* O157:H7, *Campylobacter*, and *Salmonella* in food animals on farms (Table 1). In most studies, a single phage or a cocktail of phages was orally administered to the animal. In finisher broiler chickens, a ≥2 log reduction in *Campylobacter* or *Salmonella* at different phage dosages (10^6^–10^12^ PFU/mL) was obtained (Table 1), which can contribute to a lower microbial load entering the processing stage. Except for the studies of Kittler et al. [101], Clavijo et al. [102], and Arthur et al. [103] that were performed in commercial farms and lairage targeting *C. jejuni*, *Salmonella*, and *E. coli* O157:H7, respectively, other studies (*n* = 12) concentrated on germ-free small-scaled in vivo controlled experiments. For example, the prevalence of bacteria among individuals in the natural environment is due to the level of natural exposure to the pathogen. In contrast, germ-free small-scaled controlled experiments deliver an equal dose to individuals at a preselected site within the digestive tract. Even though a challenge dose can be used to establish a baseline of phage-mediated pathogen reduction [104], differential pathogen colonization amongst individuals, differences in immune responses, nutrient availability, and the composition of the intestinal microbiome should be considered [105]. Second, a mid-log short-term culture (≥18 h) grown under optimal growth temperature (37 °C) on nutrient broth (Luria-Bertani or tryptic soy broth) administered in germ-free small-scaled controlled settings are likely to have traits that differ from environmental strains. Third, mid-log cultures from controlled settings may lack cell variants compared to environmental strains that are exposed to fluctuations in temperature and moisture. In this context, the efficacy of phage therapy is likely to be overestimated in controlled settings as mid-log growing cultures are known to be susceptible to phages [106] as compared to natural settings where cell variants may compromise phage efficacy.

Phage applications in either human therapeutic or agricultural sectors require a better comprehension of phage stability, viability, and survivability in diverse and hostile environments [81]. Except for three of the phages used in the experimental studies (Table 1), all were members of the *Myoviridae* (*n* = 9) or *Siphoviridae* (*n* = 3) of the order *Caudovirales*. Most phages were isolated from the target animal, and although their selection may be biased due to their ease of isolation and lytic potential, they have been shown to be stable and remain infective within the target animal during treatment. Some phages from the abovementioned families are known to be highly resistant to adverse environments, such as in desert surface sand exposed to high and low temperatures [107]. Extreme environments such as this may be a rich source of phages for the biocontrol of pathogens under a variety of food processing conditions.

Most studies that have used phages to control *L. monocytogenes* in food animals have focused on ready-to-eat beef, pork, and poultry. These foods typically undergo a period of cold storage which can enrich *L. monocytogenes* if it is present [20]. Moreover, a case–control study by Nightingale et al. [108] showed that *L. monocytogenes* can also proliferate in cattle, as levels were much higher in feces than in feed or the farm environment. Silage has been shown to be a common source of *L. monocytogenes* and targeting this source with phages may be an effective means of preventing on-farm transmission. Targeting feed can be more cost-effective as opposed to the direct control of *L. monocytogenes* in the host animal. However, the phage/cocktail should be selected with respect to its ability to withstand a wide range of temperatures and pH for a maximum efficacy that encompasses both the conditions in silage and those in the intestinal tract of the host animal.

**Table 1:** A summary of studies of bacteriophages used to control foodborne pathogens in/on food animals.

Target Animal	Target Bacteria	Phage/Family	Phage/Mixture	Phage Dose	Phage Delivery Route	Settings	Efficacy	Reference
24-day-old broiler chicken	*C*. *jejuni*	CP20 and CP30A/*Myoviridae*	Cocktail	10^7^ PFU/mL	Oral	In vivo controlled	A reduction of up to 2.4 log_10_ CFU/g 2 days post-treatment	[94]
9-day-old broiler chicken	*C*. *jejuni*	NCTC12673, 12674, 12678, and 12672/Nd^1^	Single and cocktail	10^7^ PFU/mL	Oral	In vivo controlled	A 2.8 log_10_ CFU/g reduction 21 days post-treatment	[109]
36-day-old commercial broiler chicken	*C*. *jejuni*	NCTC12672, 12673, 12674, and 12678/*Myoviridae*	Cocktail	7.2 and 7.9 PFU/mL	Oral	Commercial farm	A 3.2 log_10_ CFU/g reduction at slaughter	[101]
25-day-old broiler chicken	*C*. *jejuni*	CP220/*Myoviridae*	Single	10^7^ and 10^9^ PFU/mL	Oral	In vivo controlled	A 2.0 log_10_ CFU/g reduction 2 days post-treatment	[110]
Sheep	*E*. *coli* O157:H7	CEV1 and CEV2/*Siphoviridae*	Single and cocktail	10^11^ PFU/mL	Oral	In vivo controlled	Cocktail had 99.9% reduction compared to 99% in single 2 days post inoculation	[111]
One-day-old Ross broiler chicks	*C*. *coli* and *C*. *jejuni*	phiCcoIBB35, phiCcoIBB37 and phiCcoIBB12/*Myoviridae*	Cocktail	10^6^ or 10^7^ PFU/mL	Oral	Commercial farm	A 2.0 log_10_ CFU/g reduction 2 days post-treatment	[112]
16-month- and 8–9-year-old cattle	*E*. *coli* O157:H7	e11/2 and e4/1c/*Myoviridae*	Cocktail	10^11^ PFU/mL	Oral	In vivo controlled	No significant difference compared to control 2 days post inoculation	[113]
≥1-year-old cattle (steer)	*E. coli* O157:H7	rV5, wV7, wV8, and wV11/Nd^1^	Cocktail	10^10^ PFU/bolus and 10^11^ PFU/feed	Oral bolus or phage mixed in cattle feed	In vivo controlled	The duration of shedding was reduced by 14 days in bolus-fed steers as compared with control steers, but phage did not reduce *E*. *coli* O157:H7 shedding overall	[99]
Cattle before passing through the lairage	*E. coli* O157:H7	Finalyse^®^/Nd^1^	Cocktail	10^10^ PFU/gallon of water	Sprayed on hide	Commercial farm	No significant reduction after 3 days of application compared to control	[103]
Cattle	*E*. *coli* O157:H7	rV5, wV7, wV8, and wV11/*Myoviridae*	Cocktail	10^11^ PFU/mL	Oral and rectal	In vivo controlled	No significant difference compared to control over 83 days post inoculation	[114]
Six-month-old Holstein steers	*E*. *coli* O157:H7	KH1 and SH1/Nd^1^	Cocktail	10^11^ PFU/mL	Recto-anal junction	In vivo controlled	Reduction in the average number of *E*. *coli* O157:H7 among phage-treated steers compared to control steers	[115]
38-day-old broiler chicken	*S*. *enterica* serotypes Enteritidis, Typhimurium, and Hadar	ϕ151/*Myoviridae*, ϕ10, and ϕ25/*Siphoviridae*	Single	10^9^ or 10^11^ PFU/mL	Oral	In vivo controlled	Phage ϕ151 had a 4.2 log_10_ CFU/g reduction 1 day post-treatment for both *S*. Enteritidis and Typhimurium. Phage ϕ10, a 2.19 log_10_ CFU/g reduction for *S*. Typhimurium. No reduction by ϕ25 on Hadar	[116]
18-day-old commercial broiler chicken	*Salmonella*	SalmoFREE^®^ (φ San15, φ San23, φ San24, and φ San25)/*Myoviridae*	Cocktail	10^8^ PFU/mL	Oral	In vivo controlled	100% reduction on day 33 post-treatment compared to control	[102]
One-day-old broiler chicken	*S*. Typhimurium	Φ*st*1/*Siphoviridae*	Single	10^10^ or 10^12^ PFU/mL	Intracloacal	In vivo controlled	100% reduction after 1 day post-treatment compared to control	[117]
4-day-old broiler chicken	*S*. *enterica* serotype Enteritidis	CNPSA1, CNPSA3, and CNPSA4/Nd^1^	Single	10^11^ PFU/mL	Oral	In vivo controlled	A reduction of 3.5 orders of magnitude of CFU/g 5 days post treatment	[118]

Nd^1^ = not determined in the studies analyzed.

### 4.2. Application of Phages for Controlling Foodborne Pathogens in Leafy Green Vegetables at Pre-Harvest Level

Vegetables are the main sources of plant fiber, minerals, and vitamins necessary for a nutritious and healthy diet. Like food-producing animals, leafy green vegetables (LGVs) such as spinach, lettuce, cabbage, and Swiss chard are potential vehicles of foodborne pathogens. Most LGVs are consumed fresh, hence increasing the risk that they may be a transmission source of pathogens involved in foodborne outbreaks [119]. STEC O157, *Salmonella*, *Campylobacter*, and *L*. *monocytogenes* are often associated with foodborne disease outbreaks through LGVs [120,121,122,123,124,125]. Contaminated farmland, manure, and irrigation water contaminated with feces from livestock and wildlife are the primary sources of LGV contamination at the farm [126,127]. Phage-based control targeting these sources of contamination has the potential to reduce the risk of LGVs being contaminated with pathogens.

To our knowledge, there is no evidence of a commercial field-scale experiment using phages for the pre-harvest control of STEC O157, *Salmonella*, *Campylobacter*, or *L*. *monocytogenes* in LGVs. LGVs are usually grown in large areas and are prone to various plausible sources of contamination, like contaminated farmland, irrigation water, and animal feces through runoff waters. Also, identifying a contaminated area of the farm and pathogen location on or in the LGVs can be challenging for localized and target application of phages [128]. Finally, spraying the entire farm with phages using irrigation water as a carrier is presently economically infeasible due to the large quantities of phages that would be required.

## 5. Food-Processing Environment

According to Rosenquist et al. [129], the incidence of *Campylobacter*-related illnesses linked with the consumption of contaminated food could be reduced 30 times if the number of pathogens on food-animal carcasses was decreased by 2 log during processing. In addition to the potential contamination of meat and LVGs during harvest, pathogens can also originate from the food processing environment, poor food handling, and contaminated food contact surfaces. Using phage/cocktails to kill pathogens in the food processing environment and on food contact surfaces before and after food processing could substantially reduce the risk of food contamination.

Several studies have used phages to control STEC O157 and non-O157, *Salmonella*, *Campylobacter*, and *L*. *monocytogenes* in food processing environments. Reinhard et al. [130] used a *Listeria*-specific phage to successfully reduce *Listeria* populations in a processing plant (Table 2). To our knowledge, data using this approach to control resident environmental foodborne pathogens are limited. It is possible that resident foodborne pathogens in the processing plant on non-food contact surfaces can contaminate food products through the generation of aerosols [131]. Control measures that target resident pathogen strains are necessary because they can lower the pathogen heterogeneity at the processing level and subsequent contaminations. Although controlling foodborne pathogens on non-food contact surfaces can be promising, it can be laborious and challenging as to where and how the phage/cocktail can be applied. This is because the applied phage/cocktail needs to encounter all surfaces within the processing plant. Also, differential colonization and growth state of pathogens within the plant may require different concentrations of phages or a cocktail for effective control. For example, it is known that actively growing bacterial cells in small numbers are eliminated by phages, whereas larger colonies survive and retain a mixture of sensitive and resistant variants [106]. Likewise, a better understanding of the host density and the extent of host–phage interaction that influences the degree of post-treatment phage proliferation is required. A chemotactic-based, target-specific engineered phage could be a means of overcoming conditions where phage efficacy is dependent on random bacteria–phage contact.

Experimentally contaminated food/food surface studies indicate that foodborne pathogens can form mono-species biofilms on food contact surfaces and that a population reduction of 1–5.4 log_10_ CFU/cm^2^ at varied temperature conditions is achievable using a single phage type or cocktails (Table 2). These findings indicate the potential of using phage/cocktails to reduce mono-species biofilm cells on food contact surfaces. However, biofilm formation in an experimentally controlled setting may not necessarily reflect that in processing plants. Also, incompletely eradicated biofilm cells can regrow after treatment [132], resulting in downstream contamination. Multiple bacterial species [133], nutrient availability [134], and temperature [135] are factors that can affect biofilm formation and vary between experimentally controlled studies and the natural environment. Therefore, their role in biofilm formation by a foodborne pathogen on food surfaces in processing plants needs to be clearly understood before phage control measures are employed. Also, a biofilm architecture consisting of an exopolysaccharide matrix and multiple bacterial layers can confer resistance [136] by affecting phage diffusion within the biofilms. This is contrary to the expectation that because phages are non-motile, they will be more efficient killers of localized than dispersed bacteria cells, as phage progeny can easily infect nearby cells. One way that phages can gain access to cells within biofilms is through the production of depolymerases [137,138] that can degrade the biofilm matrix. Another challenge to phage efficacy in biofilms is that persister cells, which are metabolically inactive within biofilms, lack the resources like amino acids, ATP, polymerases, and ribosomes that phages require for replication [139]. In this context, phages can infect these non-growing cells but cannot produce progeny for further infection of additional target cells. In a scenario where non-growing cells are infected by phages, it can be dubbed ‘held at gunpoint’, as the reactivation of cell growth is on approach to triggering phage activity [140]. Though phage-based application is a potential method for controlling foodborne pathogens, identifying and optimizing phages for target eradication of biofilms may call for substantial research and development efforts. Genetically engineered phages that are specifically developed to combat biofilms may be integral to the success of phage therapy in food production environments [141].

**Table 2:** Summarized studies on bacteriophages used to control foodborne pathogens in food processing environments and on food contact surfaces.

Environment or Surface Type	Target Bacteria	Phage/Family	Phage/Mixture	Phage Dose	Bacteria Dose	Mode of Application	Temperature Condition	Efficacy	Reference
Ready-to-eat food manufacturing area (door frames/seals, floors/curbing, wheels/casters, walls/windows/curbing, drains, catch pan, water pipe, freezer doors/door seals, etc.)	*L*. *monocytogenes*	PhageGuard Listex™	Cocktail	10^7^ and 10^8^ PFU/mL	Not indicated	Spraying	4 and 20 °C	Moderate application of 10^7^ resulted in a 66% reduction in listeria prevalence at both 4 and 20 °C, whereas at concentration 10^8^, a reduction of 43 and 32% was obtained at 4 and 20 °C, respectively.	[130]
Glass	*C. jejuni* (NCTC 11168 and PT14)	CP8 and CP30	Single	10^6^ or 10^9^ PFU/mL	10^5^ CFU/mL (initial cells for biofilm formation)	Spot inoculation	37 °C	A 1 to 3 log_10_ CFU/cm^2^ reduction 24 h after phage treatment compared with control.	[142]
Stainless steel and polyurethane thermoplastic belting	Cocktail of *L*. *monocytogenes* and *L*. *innocua*	P100	Cocktail	10^7^ and 10^8^ PFU/cm^2^	10^4^–10^5^ CFU/cm^2^	Spot inoculation	4 and 20 °C	Overall, a reduction of 1.27–3.33 and 1.17–2.76 log_10_ CFU/cm^2^ on stainless steel and polyurethane thermoplastic belting, respectively, with a higher reduction at a high phage dilution of 10^8^.	[143]
Spinach harvester blade	Cocktail of *E*. *coli* O157:H7	Phages not specified	Cocktail	10^8^ PFU/mL	10^5^–10^6^ CFU/mL	Spraying	22 °C	Reduction in biofilm populations by 4.5 log_10_ CFU on blades after 2 h of phage treatment.	[144]
Stainless steel	*L. monocytogenes* (19CO9, 19DO3 and 19EO3)	LiMN4L, LiMN4p, and LiMN17	Single or cocktail	10^9^ PFU/mL	10^8^ CFU/mL	Immersion	15 °C	Single phages reduced biofilm cells by 3–4.5 log units and cocktail by 3.8–5.4 and log_10_ CFU/cm^2^.	[65]
Stainless steel, rubber, and MBEC biofilm devices	*S*. Enteritidis (ATCC13076) and *S.* Typhimurium (ATCC14028)	BP 1369 and BP 1370/*Myoviridae* and *Podoviridae*, respectively.	Single	10^8^ PFU/mL	Initial inoculum of 10^5^ CFU/mL	Immersion	10 and 30 °C	A reduction in biofilm cells by 3.0, 2.0, and 3.0 log CFU/cm^2^ on stainless steel, rubber, and an MBEC device.	[145]
Stainless steel chips, ceramic tile chips, and high-density polyethylene chips	Cocktail of O157:H7 (EK27, ATCC 43895, and 472)	BEC8	Cocktail	10^6^ PFU/mL	10^6^, 10^5^, and 10^4^ CFU/chip	Spot inoculation	4, 12, 23, and 37 °C	No biofilm survivors were detected (detection limit 10 CFU/chip) after 1 h of treatment at 12, 23, and 37 °C.	[146]
Polystyrene and stainless steel	*S*. Enteritidis	PVP-SE	Single	MOIs (0.1, 1, and 10)	10^4^ CFU/mL	Immersion	4 and 22 °C	A 2–5 log_10_ CFU/cm^2^ reduction with a higher killing efficiency at room temperature.	[147]
Stainless steel	*E*. *coli* O113:H21 and O154:H10	SA21RB	Single	10^13^ PFU/mL	10^6^ and 10^5^ CFU/mL, respectively	Immersion	22 °C	A reduction in biofilm cells by 2.5 and 2.1 log_10_ CFU/cm^2^ for O113:H21 and O154:H10, respectively, for 24 h biofilm after 3 h of phage treatment.	[68]
Polystyrene microplate	*S.* Enteritidis	CW1, CW11, M4, and M10	Cocktail	Not indicated	10^2^ CFU/mL for developing biofilm. Mature biofilm (48 h) number not indicated	Immersion	37 °C	A reduction in cells in the developing biofilm and mature biofilm by 0.79 and 0.4 log_10_ CFU/cm^2^, respectively.	[148]

Food product types, available nutrients, moisture content, and temperature abuse during food processing can promote pathogen proliferation, increasing the risk of further pathogen presence in ready-to-eat (RTE) foods. Control interventions on food products can reduce colonization and the downstream flow of foodborne pathogens. Chlorine is commonly used to reduce pathogens during the wash stage in food processing plants. However, there are concerns that chlorine can inactivate phages [143]. In contrast, Ding et al. [149] found that residual chlorine on lettuce leaves did not reduce the lytic potential of *E*. *coli* O157:H7 phages. This may suggest that the effect of residual chlorine on phages needs to be evaluated before both approaches are employed within food processing environments. The isolation and identification of sanitizer-resistant phages could facilitate the synergistic application of chlorine and phages. Also, the application of phages on food/food contact surfaces before the wash step is important for the desired outcome [130].

A number of experimental studies have used phages to control STEC O157 and non-O157, *Salmonella*, *Campylobacter*, and *L*. *monocytogenes* in food products and leafy green vegetables (Table 3). These results from both commercial and non-commercial phages are promising, with log reduction (0.39–4.54) after phage applications at different temperatures (−20 to 30 °C), dosages (10^7^–10^10^ PFU/mL), bacterial densities (low level 10^2^–10^4^ CFU/mL, and high level 10^5^–10^7^ CFU/mL), time points, application method, and duration of phage exposure (5 min to 7 days). These are important factors for consideration during phage treatment. For example, phage-resistant mutant strains can develop after a longer exposure time [150], whereas phage effectiveness can also be affected by shorter exposure times if it has a long infection cycle. Spraying phages on food is generally less effective than if the food is immersed in a phage solution [151]. This response has been attributed to increased Brownian motion with immersion than spraying. Second, phage efficacy is influenced by the host–concentration [106] and is also temperature-dependent [152], with a greater reduction in the numbers of target bacteria when they are present at lower concentrations and at 21 °C than at 4 °C. However, phage activity has been reported to be active against *Salmonella* associated with chicken meat at −20 °C within the first 24 h of storage [153]. This suggests that phages need to be isolated and characterized for use at a specific temperature to optimize their efficacy against targeted pathogens. Additional factors, including experimental design, food type, bacterial host, and phage type, may influence the efficacy of the phage, requiring a full understanding of these factors in relation to the targeted pathogen.

## 6. Future Perspective and Conclusions

For a successful and sustainable strategy, phages should be used for customized treatments, as suggested by Torres-Barceló [169]. Here we propose the ‘niche-specific’ use of phages in the food processing system for effective control of pathogens, bearing in mind that a phage/cocktail against a target pathogen across the food chain may not be suitable in different niches such as the animal, plant, or food processing environments. With the knowledge of the targeted pathogen and the nature of its variants in specific environments, niche-specific phages or cocktails could be formulated for spraying or immersion and oral or topical application. More commercial field-scale studies are required, even though they are more difficult and costly than in vivo laboratory experiments, to affirm the efficacy of phages in more real-world food production systems. Such a strategy could pave the way for effective target-specific phage formulation as an additional processing aid to the ongoing efforts to further improve food safety.

## Figures and Tables

**Table 3:** Summarized studies on bacteriophages used to control foodborne pathogens in food products and on leafy green vegetables.

Food Type	Target Bacteria	Phage/Family	Phage/Mixture	Phage Dose	Bacteria Dose	Mode of Application	Temperature Condition	Efficacy	Reference
Beef	*E. coli* O157	EP75 and EP335	Cocktail	10^7^ or 10^8^ PFU/cm^2^	10^5^ CFU/cm^2^	Spot inoculation	4 °C	Reductions of 0.8–1.1 log_10_ CFU/cm^2^ and 0.9–1.3 log_10_ CFU/cm^2^, respectively.	[154]
Raw meatball	*E*. *coli* O157:H7	M8AEC16	Single	10^10^ PFU/mL	10^2^, 10^4^ and 10^6^ CFU/g	Immersion	4 °C	A reduction of 0.69–2.09 log_10_ CFU/g after 5 h of application.	[155]
Beef and lettuce	*E. coli* O157:H7	EcoShield™	Cocktail	10^9^ PFU/mL	10^3^ CFU/g	Spray	4 °C	Reduced the level of bacteria by ≥94% and 87% after 5 min contact time in meet and lettuce, respectively.	[156]
Beef	*E*. *coli* O157:H7	PS5/*Myoviridae*	Single	10^10^ PFU/mL	10^7^ CFU/mL	Spot inoculation	4 and 24 °C	A 2.4 log_10_ CFU/piece after 24 h post application at 4 °C, whereas a 3.5 log_10_ CFU/piece after 6 h post application at 24 °C.	[157]
Chicken	*S*. Typhimurium	PS5/*Myoviridae*	Single	10^10^ PFU/mL	10^7^ CFU/mL	Spot inoculation	4 and 24 °C	A 1.2 log_10_ CFU/piece after 24 h post application at 4 °C and a 1.6 log_10_ CFU/piece after 6 h post application at 24 °C.	[157]
Beef (coarse and fine ground)	*S*. *enterica* (ATCC 51741), *S.* Heidelberg (ATCC 8326), *S.* Newport (ATCC 27869), and *S.* Enteritidis C (Se 13)	Salmonelex™ (S16 and the FO1a)/*Myoviridae*	Cocktail	10^8^ and 10^9^	10^4^ CFU/g	Spot inoculation	5 °C	Overall, a reduction of 1.6 log_10_ CFU/g was observed after the application of 10^9^ phage.	[158]
Ground red meat trim and poultry	*S.* Infantis (ATCC 51741), *S.* Heidelberg (ATCC 8326)*, S.* Newport (ATCC 27869), and *S.* Enteritidis (SE13)	Salmonelex™ (S16 and the FO1a)/*Myoviridae*	Cocktail	10^7^ and 10^8^	10^7^ CFU/g	Tumbling	4 °C	Overall, phage application on trim reduced 0.8 and 1 log_10_ CFU/g of *Salmonella* in ground pork and beef, respectively, whereas a reduction of 0.9 and 1.1 log_10_ CFU/g occurred in ground turkey and chicken, respectively.	[66]
Chicken skin	Cocktail of *S*. Typhimurium, *S*. Heidelberg, and *S*. Enteritidis	SalmoFresh™	Cocktail	10^9^ PFU/mL	10^3^ CFU/g	Immersion in water followed by spot inoculation and in chlorine (30 ppm) followed by phage treatment	4 °C	A reduction of 0.9–1 log_10_ CFU/cm^2^ with phage only. Whereas a greater reduction of 1.6 and 1.8 log_10_ CFU/cm^2^ after 2 and 24 h. after chlorine and phage treatment.	[159]
Chicken	*S*. Typhimurium, *S*. Newport, *S*., and Thompson	Salmonelex™	Cocktail	10^7^ PFU/cm^2^	10^4^ CFU/cm^2^	Spread in sterile filtered water or sterile tap water	4 °C	A reduction of 0.39 log_10_ CFU/cm^2^ and 0.67 log_10_ CFU/cm^2^ after 30 min and 8 h post-inoculation, respectively.	[104]
Meat	*L*. *monocytogenes*	Halal-certified List-shield		10^9^ PFU/mL	Concentration not indicated	Spot inoculation	4 °C	A reduction of 2.3 log_10_ was recorded in phage-treated beef samples during the storage period of 15 days.	[160]
Fresh salmon meat	*L*. *monocytogenes*	SH3-3/*Myoviridae*	Single	10^8^ CFU/mL	10^5^ CFU/g	Spot inoculation	4 °C	A reduction of 2.67, 4.14, and 4.54 log_10_ after 24, 48, and 72 h of phage addition, respectively.	[161]
Chicken	Cocktail of *L*. *monocytogenes* strains ATCC 19113, ATCC19115, and ATCC 13932	ListShield	Cocktail	10^8^ log CFU/g	10^4^ CFU/g	Spraying	4 °C	A mean reduction of 0.56, 0.84, 0.46, and 0.10 log cycles in viable counts was observed at 0, 24, 48, and 72 h after phage treatment, respectively.	[162]
Cooked turkey and roast beef	A cocktail of *L*. *monocytogenes* (serotypes; 1/2a, 1/2b, and 4b)	LISTEX™P100	Cocktail	10^7^ PFU/cm^2^	10^3^ CFU/cm^2^	Smearing	4 and 10 °C	An initial reduction of 2.1 and 1.7 log_10_ CFU/cm^2,^ respectively, for cooked turkey and roast beef at 4 °C, while an initial reduction of 1.5 and 1.7 log_10_ CFU/cm^2^, at 10 °C.	[163]
Raw chicken and pork meat	*C*. *jejuni* (NCTC 11168) and *C*. *coli* (NCTC 12668)	NCTC group II phage 12684 or CP81	Single	MOI of 10 or 100	10^6^ CFU/mL	Spot inoculation	4 and 37 °C	No reduction at 4 °C after 7 days of inoculation.	[164]
Raw and cooked beef	*C*. *jejuni*	Cj6/*Myoviridae*	Single	MOI of 10 or 10,000	Low cell density of <100/cm^2^ or high cell density of 10^4^ CFU/cm^2^	Spot inoculation	5 and 24 °C	No reduction at 5 °C compared to control with low MOI. However, a 2 log_10_ CFU/cm^2^ reduction on raw and cooked meat at high host density and a high MOI of 10,000.	[165]
Chicken	*C. jejuni* (NCTC12662 or RM1221)	F356 and F357	Cocktail	10^7^ PFU	10^4^ CFU/cm^2^	Spot inoculation	5 °C	A 0.73 log_10_ reduction at 5 °C after 24 h post-treatment.	[67]
Chicken liver	*C*. *jejuni* (HPC5 and 81–176)	Phages ϕ3 or ϕ15/*Myoviridae*	Single	10^8^ PFU/g	10^3^ or 10^5^ CFU/g	Phage added to liver stomachates containing *C*. *jejuni*	4 °C	A 0.2 to 0.7 log_10_ CFU/g reduction 48 h post-treatment.	[166]
Lettuce	*Salmonella* ser. Enteritidis (ATCC13076) and *Salmonella* ser. Typhimurium (ATCC14028)	BP 1369 and BP 1370/*Myoviridae* and *Podoviridae*, respectively	Single	10^8^ PFU/mL	10^6^ CFU/mL	Immersion	10, 20, and 30 °C	A reduction of >1.0 log_10_ CFU/cm^2^ after 2 h of post-treatment.	[145]
Romaine lettuce	Individual strains of STEC (EDL933; O157:H7, SN061; O26: H11, SN576; O111:NM and SN608; and O103:H2)	VE04, VE05, and VE07	Single	10^8^ PFU/mL	10^7^ CFU/mL	Spot inoculation and spreading with pipet	10 °C	A reduction of 2.6–6 log_10_ CFU/cm^2^ after 3 days of storage at a temperature of 10 °C.	[167]
Romaine lettuce, mung bean sprouts, and seeds	Cocktail of *Salmonella* strains (Newport, Braenderup, Typhimurium, Kentucky, and Heidelberg	SalmoFresh™/*Myoviridae*	Cocktail	10^8^ PFU/mL	10^5^ CFU/mL	Spraying or immersion	2, 10, and 25 °C	Overall reduction by spraying SalmoFresh™ onto lettuce and sprouts reduced *Salmonella* by 0.76 and 0.83 log_10_ CFU/g, respectively, whereas a reduction of 2.43 and 2.16 log_10_ CFU/g by immersion was observed on lettuce and sprouts, respectively.	[168]
Romaine and iceberg lettuce	*E*. *coli* O157:H7	AYO26, AXO111, AXO121, AYO145A/*Myoviridae*, AXO103, AKFV33/*Siphoviridae*, and AXO45B	Cocktail	>10^8^ PFU/mL	High (10^5^ CFU/g) and low (10^3^ CFU/g)	Immersion	2 °C	A reduction of 2.6–3.2 and 1.7–2.3 log_10_ CFU/g for low and high contamination, respectively.	[149]

## Data Availability

The data presented in this study are available on request from the corresponding author.
